# Comparison of Laser-Synthetized Nanographene-Based Electrodes for Flexible Supercapacitors

**DOI:** 10.3390/mi11060555

**Published:** 2020-05-30

**Authors:** Francisco J. Romero, Denice Gerardo, Raul Romero, Inmaculada Ortiz-Gomez, Alfonso Salinas-Castillo, Carmen L. Moraila-Martinez, Noel Rodriguez, Diego P. Morales

**Affiliations:** 1 Pervasive Electronics Advanced Research Laboratory, University of Granada, 18071 Granada, Spain; raulromeromad@correo.ugr.es (R.R.); noel@ugr.es (N.R.); 2 Department of Electronics and Computer Technology, University of Granada, 18071 Granada, Spain; 3 Parque de Innovación Tecnológica, Facultad de Ciencias Físico Matemáticas, Universidad Autónoma de Sinaloa, 80040 Culiacán, Mexico; denice.gerardo@uas.edu.mx (D.G.); cmorailam@uas.edu.mx (C.L.M.-M.); 4 Department of Analytical Chemistry, Faculty of Science, University of Granada, 18071 Granada, Spain; inmaog@ugr.es (I.O.-G.); alfonsos@ugr.es (A.S.-C.); 5 Biochemistry and Electronics as Sensing Technologies Group, University of Granada, 18071 Granada, Spain

**Keywords:** flexible electronics, graphene oxide, laser-induced graphene, laser-scribing, supercapacitors

## Abstract

In this paper, we present a comparative study of a cost-effective method for the mass fabrication of electrodes to be used in thin-film flexible supercapacitors. This technique is based on the laser-synthesis of graphene-based nanomaterials, specifically, laser-induced graphene and reduced graphene oxide. The synthesis of these materials was performed using two different lasers: a CO_2_ laser with an infrared wavelength of λ = 10.6 µm and a UV laser (λ = 405 nm). After the optimization of the parameters of both lasers for this purpose, the performance of these materials as bare electrodes for flexible supercapacitors was studied in a comparative way. The experiments showed that the electrodes synthetized with the low-cost UV laser compete well in terms of specific capacitance with those obtained with the CO_2_ laser, while the best performance is provided by the rGO electrodes fabricated with the CO_2_ laser. It has also been demonstrated that the degree of reduction achieved with the UV laser for the rGO patterns was not enough to provide a good interaction electrode-electrolyte. Finally, we proved that the specific capacitance achieved with the presented supercapacitors can be improved by modifying the in-planar structure, without compromising their performance, which, together with their compatibility with doping-techniques and surface treatments processes, shows the potential of this technology for the fabrication of future high-performance and inexpensive flexible supercapacitors.

## 1. Introduction

Flexible electronics are expected to bring out a revolution in diverse fields of technology, such as electronic skin [1,2], robotics [3,4] or health-monitoring devices [5,6,7], among others. Most of the recent advances in this context have been possible due to the emergence of new conductive and flexible materials, many of which have reported outstanding results in terms of electrical conduction and integration, such as the polycrystalline silicon (poly-Si) [8,9] or several semiconducting metal oxides (e.g., SnO_2_, TiO_2_, ZnO or ITO) [10,11,12]. However, due to the complexity of their synthesis or the expensive fabrication processes required to obtained large-area samples, research groups are still exploring different alternatives that enable an inexpensive and massive fabrication of flexible electronics devices.

Therefore, the efforts in this direction have led to extensive investigations on the use of several classes of nanomaterials with different conductivity and sensing capabilities, including carbon nanotubes (CNTs), graphene-derived materials, metal nanowires or conductive polymers [13]. All these materials have in common being compatible with printing techniques, which enable their economical and efficient processing on diverse flexible substrates, thereby providing a commercially attractive possibility to obtain multifunctional electronics over large areas [14]. Thus, techniques such as screen-printing or inkjet printing have enabled large achievements in all areas involved in the development of flexible electronics devices, extending from transducers to the antennas for the wireless data transmission [15,16,17,18,19].

However, apart from the latter, novel flexible electronics applications are also demanding flexible energy storage devices that, together with energy harvesting technologies, contribute to the development of self-powered devices and, thus, to the paradigm of the ubiquitous sensing [20]. In this respect, supercapacitors are expected to play an important role, thanks to the higher power density induced by their fast charging/discharging rates when compared to conventional batteries. Supercapacitors can be classified mainly into two different groups, electrochemical double-layer capacitors (EDLCs) and pseudocapacitors, depending on the storage mechanism, i.e., the interaction between electrode-electrolyte [21]. EDLCs are those whose electrode material is not electrochemically active, and therefore the capacitance is associated with the pure physical charge accumulation at the electrode/electrolyte interface. On the contrary, the energy storage in pseudocapacitors relies on fast and reversible faradaic redox reactions occurring on the electrode surface [21,22,23]. For these reasons, in both cases, the electrode is a key element in the development of supercapacitors and then, many materials have been investigated for this purpose. In the case of pseudocapacitors, the most studied materials are transition metal oxides and conducting polymers, which promote the reversible faradaic-type charge transfers of these redox supercapacitors [24], whereas EDLCs electrodes are fabricated from nanoscale materials with high porosity and high surface area. In this latter case, carbon-based materials are preferred to play this role, due to their exceptionally high surface area, relatively high electrical conductivity and acceptable cost. These properties make porous carbon-based materials, such as Laser-Induced Graphene (LIG) or reduced Graphene Oxide (rGO) [25,26,27], ideal candidates for the fabrication of the electrodes. Many researchers agree that these kind of electrodes will play an important role in the supercapacitor technology, and that is why a big effort is being made to further optimizing its properties through doping [28] or surface treatments [29].

In this context, this work is focused on the study of a cost-effective technique for the synthesis of raw graphene-based EDLCs electrodes and its optimization. Following a laser-photothermal process, we synthesized electrodes based on both LIG and Laser-rGO (LrGO) using two different kind of laser beams: CO_2_ and UV. These electrodes were tested under the same conditions of layout and electrolyte to compare their performance as EDLCs electrodes. The work is structured as follows: after this introduction, Section 2 summarizes the different materials used in our experiments, as well as the methodologies followed for the fabrication and characterization of the samples. Section 3 presents the results obtained for the different electrodes together with a comparison of their performance. Finally, the main conclusions are drawn in Section 4.

## 2. Materials and Methods 

### 2.1. Materials

Two different flexibles substrates were used for the fabrication of the electrochemical capacitors, Kapton^®^ HN films with a thickness of 75 μm from DuPont Corporation (Wilmington, DE, USA) and Polyethylene Terephthalate (PET) foils with a thickness of 160 µm from ColorGATE Digital Output Solutions GmbH (Hannover, Germany). Graphene Oxide at a concentration of 0.4 wt% was acquired from Graphenea (San Sebastián, Spain). The electrolyte was prepared using poly(vinyl alcohol) (PVA, Mw 31,000–50,000, 98%–99% hydrolyzed) and phosphoric acid (H_3_PO_4_, product name: 1005731000), both acquired from Sigma-Aldrich (St. Louis, MO, USA). Electrical access to the capacitive devices was printed using a silver-based conductive paint from RS PRO (RS Components, Corby, UK).

### 2.2. Fabrication Processes

The gel electrolyte was prepared by dissolving 1 g of PVA (Mw 31,000–50,000, 98%–99% hydrolyzed, from Sigma-Aldrich, St. Louis, MO, USA) in 10 mL of de-ionized water (10 wt%), stirring at 80 °C for 2 h using a hot plate stirrer (Scilogex SCI280-Pro, from Scilogex, LLC, Rocky Hill, CT, USA). Once the PVA was completely dissolved, 1.5 mL of H_3_PO_4_ was added to the solution and it was stirred for another hour [30,31,32]. 

**Figure 1:**
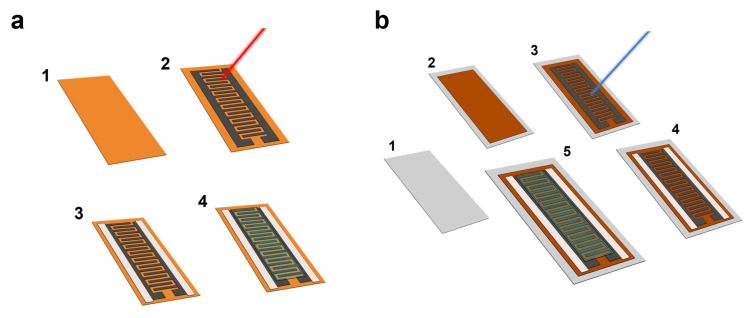
Schematic representation of the fabrication process of the flexible electrochemical capacitors (ECs). (**a**) Laser-Induced Graphene (LIG)-based ECs: (1) Kapton^®^ polyimide film (thickness: 75 µm), (2) laser-scribing process to induce graphene-derived pattern on the surface of the polyimide (this case shows the CO_2_ laser beam), (3) silver electrical contacts printed on each electrode, (4) poly(vinylalcohol) (PVA)/H_3_PO_4_ electrolyte drop-casted on top of the InterDigital Electrodes (IDE) structure. (**b**) Laser-reduced Graphene Oxide (rGO) (LrGO)-based ECs: (1) PET film (thickness: 160 µm), (2) GO deposited onto the PET substrate (concentration: 75 µL/cm^2^), (3) laser-scribing process to reduce the GO, (4) silver electrical contacts printed on each electrode, (5) PVA/H_3_PO_4_ electrolyte drop-casted on top of the IDE structure.

**Figure 2:**
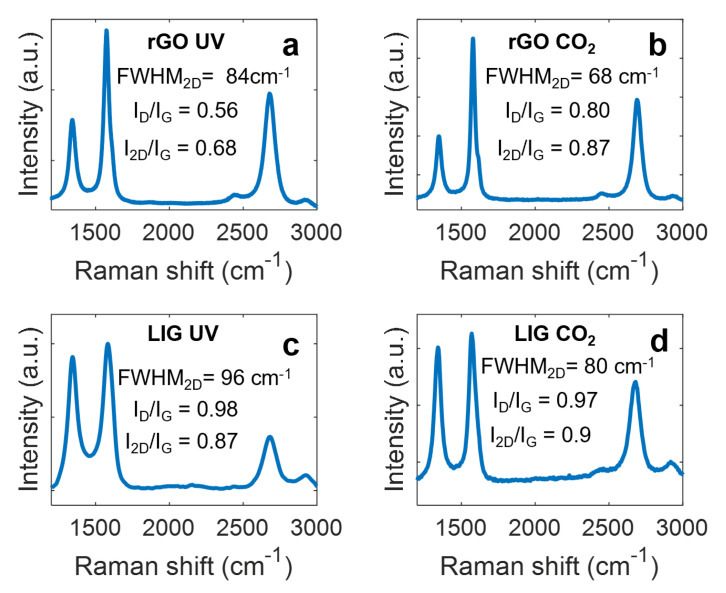
Raman spectra of the different electrodes, (**a**) rGO UV, (**b**) rGO CO_2_, (**c**) LIG UV (**d**) LIG CO_2__._ Acquisition parameters: wavelength: 532 nm, data interval: 1 cm^−1^, exposure time: 15 s, accumulations: 5, center number: 1469.99 cm^−1^. GO spectrum can be found in supplementary Figure S2.

**Figure 3:**
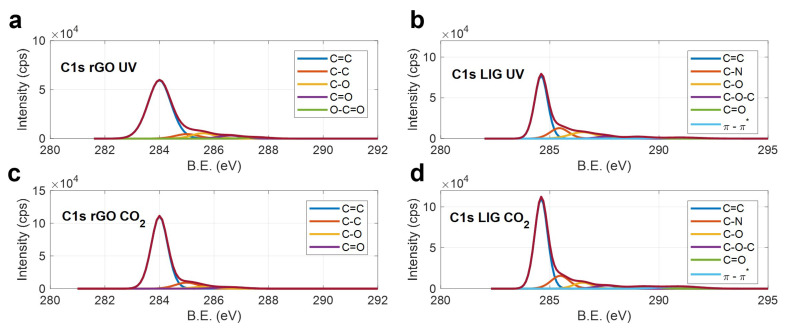
XPS C1s peaks of the different electrodes, (**a**) rGO UV, (**b**) LIG UV, (**c**) rGO CO_2_ (**d**) LIG CO_2_.

The devices were fabricated following the schemas shown in Figure 1. In the case of the LIG-based supercapacitors (Figure 1a), the laser photothermal ablation was carried out directly on the polyimide film (Figure 1(a-1)), inducing the graphene-derived structures on its surface (Figure 1(a-2)) [33,34]. On the other hand, for the fabrication of LrGO-based electrodes, a PET foil (Figure 1(b-1)) was covered with GO at a concentration of 75 µL/cm^2^ (Figure 1(b-2)). After that, the sample was dried at ambient conditions during 48 h. Once the drying process was completed, the GO was turned into rGO through the laser photothermal process, as shown in Figure 1(b-3). 

Two different types of laser were used in this work for this purpose. On one side, we used a CO_2_ laser with an infrared wavelength of 10.6 µm (Rayjet 50, from Trotec Ltd., Marchtrenk, Austria). On the other side, we also used a UV laser with a wavelength of 450 nm (from KKmoon SA, Automatic K5). The use of this type of technology requires two main safety measures. Thus, the laser machines count on with an eye protection shielding, which filters the high intensity wavelength of their corresponding laser beams. In addition to that, a fume extractor was used during the laser photothermal treatment to avoid the inhalation of the gases released during this process.

Both laser power and speed were set, in order to optimize the sheet resistance of the graphene-derived patterns, thus improving the effective electrons transport and the electrochemical property [35]. Therefore, we obtained a total of four different combinations laser-electrode (hereinafter referred to as LIG UV, LIG CO_2_, rGO UV, rGO CO_2_ for each material and laser, respectively). 

The layout studied in this work consisted of planar InterDigital Electrodes (IDE) structure (Figure 1), given that this configuration allows to achieve lower thicknesses and a more accurate control of the distances between electrodes than its stack counterpart [36]. In addition, we considered two different patterns, firstly we characterized the different devices using the following layout: number of fingers *N* = 20, width of the fingers *W* = 1 mm, spacing between electrodes *S* = 1 mm, interspacing between fingers *i* = 1 mm and length of the fingers *L* = 1 cm, which resulted in an effective area of ~4 cm^2^. Secondly, we also used a layout with the same effective area, but with different spacing between electrodes (*S* = 500 µm), interspacing between fingers (*i* = 500 µm), as well as the width of the fingers (*W* = 500 µm), to demonstrate the possibility of increasing the specific capacitance by changing the geometry of the IDE structure.

**Figure 4:**
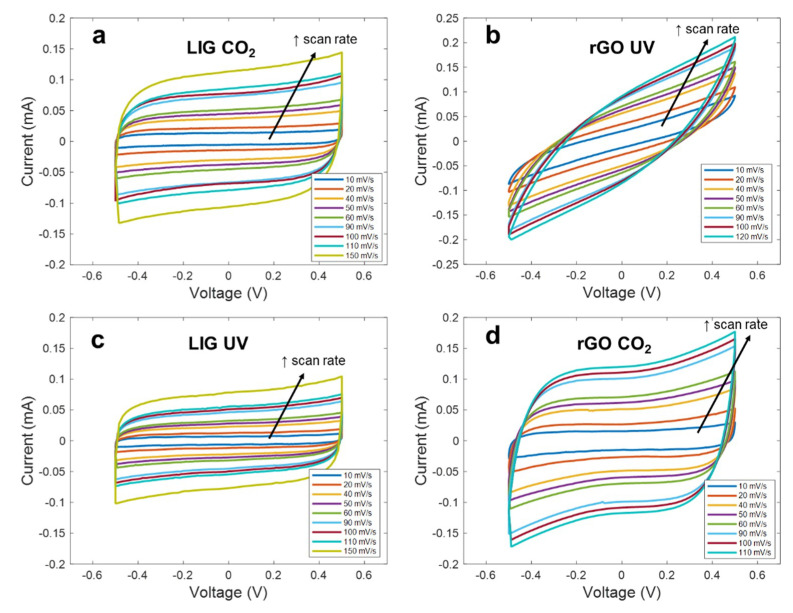
Cyclic voltammetry curves at different scan rates for the different electrodes (**a**) LIG CO_2_, (**b**) rGO UV, (**c**) LIG UV, (**d**) rGO CO_2_.

**Figure 5:**
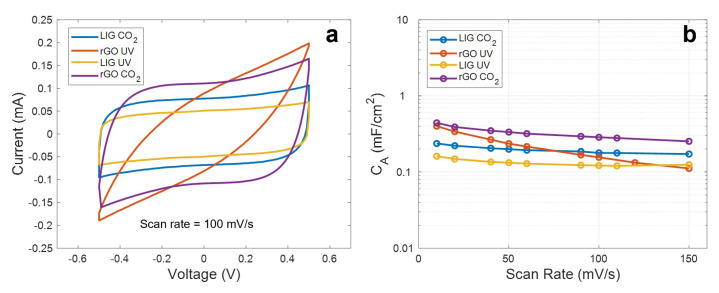
(**a**) Cyclic voltammetry curves at 100 mV/s. (**b**) Specific capacitance as a function of the scan rate.

After the laser scribing process, electrical contacts were printed on both sides using silver ink (Figure 1(a-3) and Figure 1(b-4)) with the objective of reducing the resistivity between the electrode and the current collector, and with that the Equivalent Series Resistance (ESR) of the capacitor [37,38]. Finally, ~1.5 mL of the gel electrolyte was drop-casted on top of the capacitive IDE structure covering all the effective area, as depicted in Figure 1(a-4,b-5), and the samples were left standing overnight to remove the excess of water before the characterization. 

### 2.3. Characterization

The sheet resistance of the graphene-derived patterns was extracted through the Transmission Line Method (TLM) [34]. X-ray Photoelectron Spectroscopy (XPS) analysis was performed using the Katros Axis Ultra-DLD X-ray photoelectron spectrometer (from Kratos Analytical Ltd., Manchester, UK). The samples were characterized in a vacuum chamber at a pressure of 10^−10^ Torr at an X-ray power of 450 W. Raman spectra were obtained with a NRS-5100 dispersive micro-Raman spectrometer (from JASCO International Co. Ltd., Tokyo, Japan) using an excitation source with a wavelength of λ = 532 nm (Elforlight G4-30; Nd:YAG). The profilometry of the samples was acquired using a DekTak XT contact profilometer (from Bruker Corporation, Billerica, MA, USA) at a stylus force of 1 mg. Cyclic Voltammetry (CV) experiments were performed using the B2912A precision source-measurement unit (SMU) from Keysight Technologies, Inc. (St. Rose, CA, USA).

## 3. Results and Discussion 

The feasibility of both CO_2_ and UV lasers to induce porous nanographene aggregates from commercial polyimides, and to reduce the GO, has been already proved in several works [6,28,33,34]. However, due to the different nature of the laser beams, not only is the power required to optimize the sheet resistance different, but so are the properties of the material synthetized [39]. Furthermore, the power required for the synthesis of these materials depends on the laser used, thus, we set the laser speed and studied the sheet resistance of the laser-synthetized patterns as a function of the laser power, in order to minimize the sheet resistance while maintaining the integrity of the substrates. Following the TLM procedure, whose results are summarized in Supplementary Figure S1, the configuration of the laser to minimize the sheet resistance is presented in Table 1.

As has been reported in previous works, one of the distinctive features of LIG, which is common for both UV and CO_2_ laser exposition sources, is its highly porous structure, which is a consequence of the rapid liberation of gaseous products during the laser-scribing process, also associated with the drastic increase in the atomic percentage of carbon [34,40,41,42]. In the case of the LrGO, the laser treatment on the surface of the GO leads to a partial restoration of the crystallographic network of its graphitic structure, which was disrupted during the oxidation process [43]. However, this partial recovery makes the surface of the resulting material to present structural defects manifested thorough a large roughness and a 3D plate-shape structure with multiple craters, a phenomenon that has been reported for both UV [44] and CO_2_ lasers [45], and that could be helpful to increase their double layer capacitance effect.

Raman and XPS spectroscopies confirmed the graphene-derived nature of these materials. On one hand, as seen in Figure 2, all Raman spectra are composed mainly by three different peaks located at ~1345 cm^−1^ (D peak), ~1580 cm^−1^ (G peak) and ~2700 cm^−1^ (2D peak), respectively. In particular, the G peak is associated with the sp^2^-hybrized carbon networks of the graphitic materials, whereas the D peak reveals the presence of defects in this structure (it would be inexistent in single-layer pristine graphene). Moreover, the 2D peak and its full width at half maximum (FWHM_2D_) are of particular interest for the study of the multi-layer nature of these materials [46]. 

In single-layer pristine graphene, the ratio I_2D_/I_G_ is ~2–3, and it decreases as the number of layers increase [47]. On this basis, the results of the rGO sheets showed that the rGO produced with the CO_2_ laser presents a lower number of layers (higher I_2D_/I_G_ ratio) than the one induced with the UV laser, which might be related to its lower thickness (~12 µm, in comparison with ~18 µm, according to profilometry results) due to the higher irradiation power, as demonstrated in other works [48]. In addition, the lower FWHM_2D_ also indicates that these layers have a better crystallographic structure [49]. The better recovery of the crystallographic structure of the rGO obtained with the CO_2_ laser is an indication of a more effective reduction process, which explains its smaller sheet resistance, in spite of having lower thickness. Moreover, the high I_D_/I_G_ ratio indicates that the CO_2_ laser rGO also presents a high defect density. In the case of the LIG electrodes, the higher I_D_/I_G_ ratio and FWHM_2D_ indicate a higher defective and disordered structure (i.e., lower crystallographic quality) with respect to the rGO with no significant difference between lasers sources. 

On the other hand, the XPS analysis demonstrates that the ablated surfaces are mainly composed of carbon and oxygen (present as carbon–oxygen functional groups), and that the laser-treatment led to a drastic increase in the original C/O ratio of the raw materials, as summarized in Table 2. 

The highest C/O ratios are those obtained from the LIG synthetized with the CO_2_ (C/O = 19.74) and UV (C/O = 9.45) lasers, respectively, being the CO_2_–produced samples the ones reporting a higher level of reduction. A more detailed study of the nature of these changes can be obtained by means of the analysis of the high-resolution C1s XPS spectra, the results of which are shown in Figure 3. 

As expected, the lasers are able to remove efficiently the oxygen-containing functional groups of the raw GO material (see Figure S3), as well as most of the C–N, C–O–C and C=O bonds, which compose the Kapton^®^ HN structure. For the rGO, the remaining non-desirable bonds after the laser treatment are mainly associated with carbon–oxygen compounds in the case of the UV laser (Figure 3a), and with sp^3^ hybridized carbon bonds for the rGO reduced with the CO_2_ laser (which could explain its high I_D_/I_G_ ratio). Finally, regarding the LIG, the intensity of the C=C peak, when compared with the intensity of rest of the compounds, indicates that the CO_2_ laser allowed a higher isolation of the sp^2^ hybridized atoms with respect to the LIG induced with the UV laser (Figure 3b,d, respectively), which is in accordance with its high carbon percentage.

Once all four different materials were analyzed from a structural point of view, we studied their electrochemical performance as electrodes for flexible supercapacitors. For that, we firstly performed the Cyclic Voltammetry (CV) experiments, considering a potential window of Δ*V* = 1 V (from −0.5 V to +0.5 V) at different scanning rates. The CV curves obtained for the different electrodes are shown in Figure 4. In addition, the CV curves at a scan rate of 100 mV/s are plotted together in Figure 5a, whereas Figure 5b presents the specific capacitance as a function of the scan rate extracted according to Equation (1).

(1) $$C_{A}=\frac{1}{A\cdot \Delta V\cdot s}\cdot \left(\int I\left(V\right)dV\right)$$

where *A* is the area, Δ*V* the potential window, *s* the scan rate and *I*(*V*) the current response as a function of the voltage [27].

As seen, apart from the rGO synthetized with the UV laser, the curves maintain a highly symmetric quasi-rectangular shape over the increasing scan rates, indicating a good reversible EDLC behavior and a fast charge propagation within the electrode [50]. However, it can be noted that all these curves differ from each other, indicating that the interaction electrode-electrolyte is different in each case. Thus, regarding the LIG electrodes, we can appreciate that the material synthetized with the different lasers presents an identical electrochemical behavior, but with the difference of a lower capacitance, in the case of the LIG UV electrode. This difference in capacitance is practically constant along the different scan rates (e.g., 0.23 mF/cm^2^ against 0.16 mF/cm^2^ at 10 mV/s and 0.18 mF/cm^2^ against 0.12 mF/cm^2^ at 100 mV/s), indicating that this constant difference in capacitance might be associated with the difference in the effective area of the LIG electrodes, as a result of the lower mechanical resolution of the UV laser when compared to the CO_2_ laser, which yields a lower effective area of the electrodes [40]. In the case of the rGO CO_2_ electrodes, their electrochemical behavior agrees with the reported for equivalent devices presented in the literature, such as the ones presented by Ghoniem et al. [45] and Yoo et al. [51]. It can be also noted that, even though the sheet resistance of the rGO CO_2_ electrodes is higher than the obtained for the LIG patterns, they report the best performance in terms of specific capacitance (0.44 mF/cm^2^ at 10 mV/s) as a consequence of their higher specific surface area, when compared to the LIG, which could be up to 4.5 times higher, in the case of the laser-synthetized rGO according to references [42] and [52].

Lastly, the tilted CV curves obtained with the rGO UV electrodes represent a high ESR, as a consequence of the sheet resistance of these patterns, as well as a larger internal resistance to penetrate into the pores of the electrode, as a result of their less reduced state with a high concentration of sp^3^ carbon bonds [53]. This is also reflected in the behavior of the capacitance as the scan rate increases. As seen, for low scan rates, the specific capacitance achieved with these electrodes is quite similar to that obtained with the rGO-CO_2_, since, at these rates, the ions have enough time to penetrate deeply into pores; however, it decreases gradually as the frequency increases (2 µF·s/mV), due to the reduction of the penetration depth, hence, worsening their performance [54].

**Figure 6:**
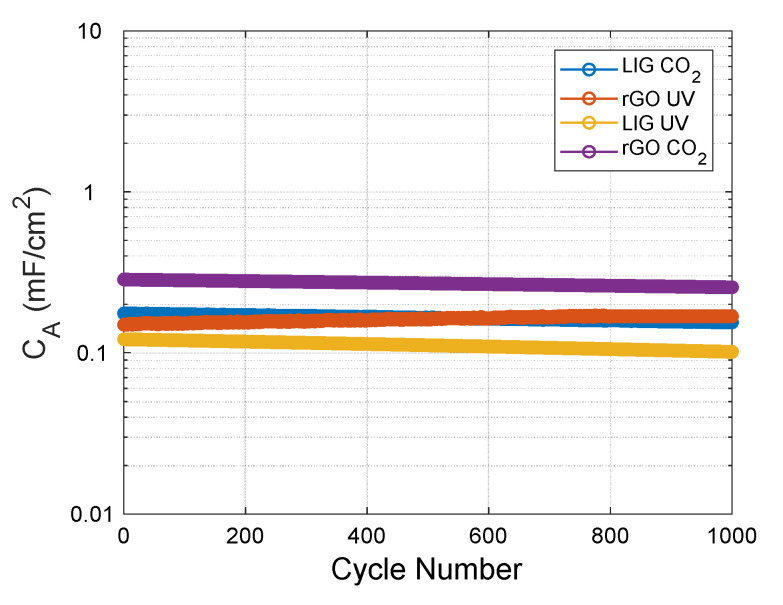
Specific capacitance as a function of increasing cyclic voltammetry cycles at a scan rate of 100 mV/s obtained with the different electrodes.

Furthermore, we tested the cyclability of the different electrodes over an increasing number of CV cycles (at 100 mV/s), extracting the specific capacitance for every single cycle (Figure 6). As observed, the capacitors are able to retain their capacitance with a variation less than 0.03 µF/cycle in all cases (being the LIG UV those with less retention capacity ~83% after 1000 cycles). As expected, the capacitance decreases as the cycles increase, with exception of the rGO UV electrodes, whose capacitance increases (up to ~700 cycles), and afterwards retains almost the same value, as a consequence of the electrochemical reduction of some of the remaining oxygen groups of these still highly-oxidized electrodes, as has been demonstrated in other works [27,55].

According to the previous results, the LIG electrodes synthetized with both lasers, as well as the rGO synthetized with the CO_2_ laser, show promising results for potentially being used as bare electrodes in supercapacitors. In particular, the LIG electrodes synthetized with the UV laser are of particular interest for the development of really inexpensive electrodes, since, using a low-cost and low-power laser, they achieve comparable results to that obtained with a CO_2_ laser, which could be further improved by increasing the mechanical resolution of CNC system driving the laser. In addition, both fabrication approaches are also compatible with the techniques of functional nanomaterials growth, as well as doping procedures in the case of the GO, which would help to improve their electrochemical activity [56,57,58].

However, it is also possible to obtain higher specific capacitances without resorting to any doping or additional treatment of the bare electrodes, simply by modifying the layout of these structures. Thus, as an example, we considered an alternative microstructure for the LIG UV and rGO CO_2_ capacitors. This structure covers the same area than the previous one but with a distance between electrodes, spacing and width of the fingers of 500 µm, instead of 1 mm.

**Figure 7:**
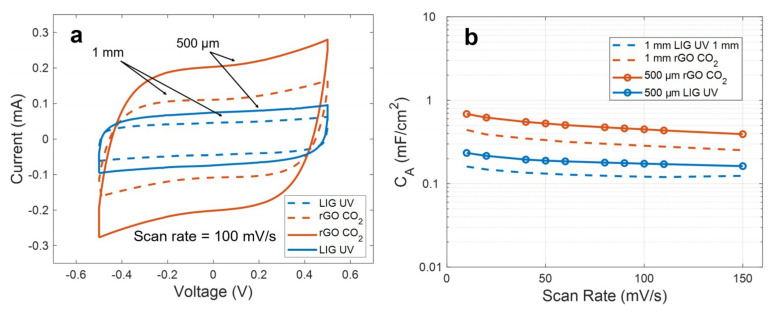
(**a**) Comparison of the cyclic voltammetry curves at 100 mV/s for two different configuration of electrodes. (**b**) Specific capacitance as a function of the scan rate for these configurations.

As a result, this structure led to an increase of the capacitance in both cases, without compromising the electrochemical performance of the devices, as can be observed in Figure 7a. This increment arises from the increase of the capacitance between two consecutive fingers by reducing their interspacing, as well as from the increment of the density of fingers, according to the IDE analytical models [59,60,61]. In our case, the increment, depicted in Figure 7b, represents a factor of 1.42 ± 0.05 for the LIG UV electrodes and of 1.73 ± 0.03 for the rGO CO_2_, resulting in a specific capacitance at 10 mV/s of ~0.23 mF/cm^2^ and ~0.7 mF/cm^2^, respectively. These values compare well to the ones reported for similar devices fabricated with other materials, such as activated carbon [62] or graphene-CNTs [63], and it is similar or even higher than the reported results for other laser-synthetized materials [64,65]. 

**Figure 8:**
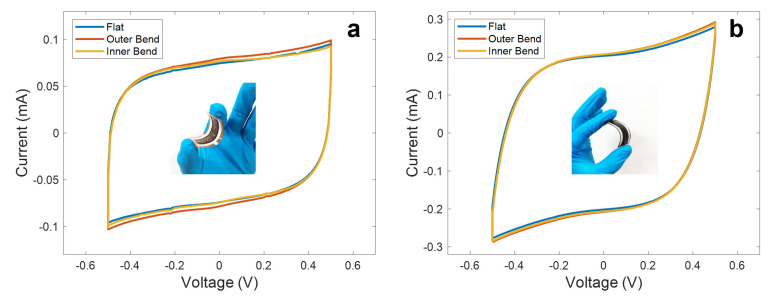
Cyclic voltammetry curves at a scan rate of 100 mV/s at different bend conditions (bend radius of 1.25 cm) obtained for the (**a**) LIG UV and (**b**) the rGO CO_2_ supercapacitors. Inset of (**a**) shows an inner bent LIG-based supercapacitor, while inset of (**b**) shows an outer bent rGO-based supercapacitor.

In addition, it is shown in Figure 8 how these devices are capable of maintaining their electrochemical performance under different bend conditions, without significant changes to their specific capacitance (being the maximum deviation with respect to the flat state ∆*C*/*C*_0_ (%) = 1.87 in the case of LIG electrodes and ∆*C*/*C*_0_ (%) = 5.45 in the case of the rGO electrodes). This maximum deviation was reported for the outer bend state in both cases. 

**Figure 9:**
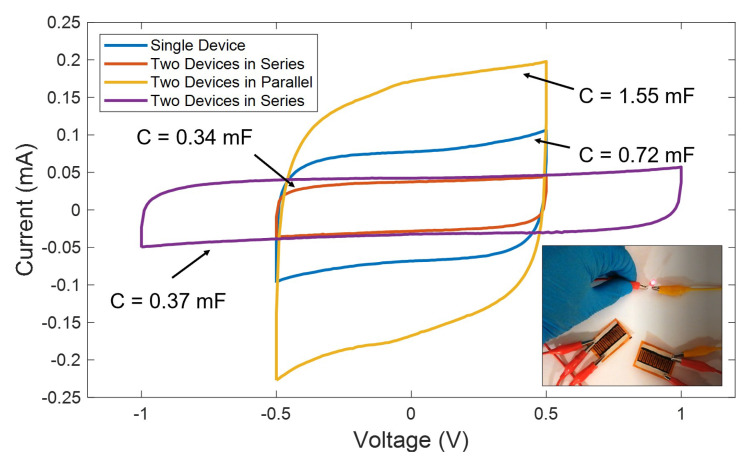
Cyclic voltammetry curves at a scan rate of 100 mV/s for the LIG UV-based supercapacitors obtained under different series-parallel configurations. Inset shows the power up of a red LED using two devices in parallel connection.

Furthermore, in applications where the size is not a constraint, it is always possible to combine different supercapacitors to increase either the current delivered or the operation voltage, as represented in Figure 9. For instance, here we present the CV curves of a single LIG UV supercapacitor, together with different combinations of two devices at a scan rate of 100 mV/s. As seen, when two supercapacitors are combined in series, they operate at double of the voltage, without compromising their performance at the cost of an equivalent capacitance, which is a half of the presented by a single device. Alternatively, when they are connected in parallel, the equivalent is twice the capacitance of a single device, hence, duplicating the nominal current. Finally, to show a practical application, we used a parallel configuration of two devices to power up a red LED bulb (as shown in the inset of Figure 9). For that, the cell was previously charged at 1 mA up to reach 2.5 V.

## 4. Conclusions

In this work, we reported a comparative study of the laser synthesis of graphene-based electrodes to be used in flexible electric double-layer capacitors (EDLCs). We considered two different kind of lasers, a CO_2_ laser with an infrared wavelength of λ = 10.6 µm and a low-power UV laser (λ = 405 nm) to synthetize laser-induced graphene from Kapton^®^ HN polyimide films, as well as to reduce a graphene oxide layer deposited onto a PET foil. After the optimization of the parameters of the lasers to minimize the sheet resistance of the laser-synthetized patterns, we studied the resulting materials and their performance as electrodes for electric double-layer capacitors. The experiments showed that the LIG electrodes synthetized with the low-cost UV laser are able to provide a specific capacitance that compares well to that obtained with the CO_2_ laser. Furthermore, it has been demonstrated that, under similar conditions, the rGO electrodes fabricated with the CO_2_ laser make it possible to obtain the highest specific capacitance, whereas the reduction achieved with the UV laser was not enough to provide a good electrochemical performance, given the high degree of oxidation of the resulting material, which increases the equivalent series resistance of the capacitive structure, as well as the motion resistance of electrolyte ions within the pores of the electrode. Finally, we proved how, by reducing the width of the fingers and the distance between them, hence, increasing the density of fingers, it is possible to enhance the specific capacitance of the these EDLCs. 

The authors believe that the technology presented in this work contributes to the study of bare electrodes for the fabrication of inexpensive flexible supercapacitors, while further studies aim to extend the comparison in combination with doping techniques. 

## Figures and Tables

**Table 1:** Values of the sheet resistance obtained after optimization for the different laser beams and materials.

Laser	Material	Laser Power (W)	Laser Speed (cm/s)	Sheet Resistance (Ω/sq.)
**CO_2_**	rGO	1.5	15	196.8
	LIG	6	15	43.3
**UV**	rGO	0.39	1	305.7
	LIG	1.5	1	240.1

**Table 2:** Carbon and oxygen atomic concentrations their ratio obtained extracted from the X-ray Photoelectron Spectroscopy (XPS) spectra for the different samples.

Material	Carbon Content (%)	Oxygen Content (%)	C/O Ratio
**Kapton^®^**	78	18	4.33
**LIG-CO_2_**	95.72	4.85	19.74
**LIG-UV**	87.72	9.28	9.45
**GO**	68.73	29.85	2.30
**rGO-CO_2_**	87.42	9.83	8.89
**rGO-UV**	84.45	10.70	7.89

## Supplementary Materials

The following are available online at https://www.mdpi.com/2072-666X/11/6/555/s1, Figure S1: Resistance as a function of the distance between consecutive contacts extracted from the TLM measurements, Figure S2: Raman spectra of the graphene oxide. Acquisition parameters: wavelength: 532 nm, data interval: 1 cm^−1^, exposure time: 15 s, accumulations: 5, center number: 1469.99 cm^−1^, Figure S3. XPS C1s peaks of the graphene oxide.

## References

[1] Liu Y., Pharr M., Salvatore G.A. (2017). Lab-on-Skin: A Review of Flexible and Stretchable Electronics for Wearable Health Monitoring. ACS Nano.

[2] Ho D.H., Sun Q., Kim S.Y., Han J.T., Kim D.H., Cho J.H. (2016). Stretchable and Multimodal All Graphene Electronic Skin. Adv. Mater..

[3] Ntagios M., Nassar H., Pullanchiyodan A., Navaraj W.T., Dahiya R. (2019). Robotic Hands with Intrinsic Tactile Sensing via 3D Printed Soft Pressure Sensors. Adv. Intell. Syst..

[4] Navaraj W.T., Núñez C.G., Shakthivel D., Vinciguerra V., Labeau F., Gregory D.H., Dahiya R. (2017). Nanowire FET Based Neural Element for Robotic Tactile Sensing Skin. Front. Neurosci..

[5] Wang X., Liu Z., Zhang T. (2017). Flexible Sensing Electronics for Wearable/Attachable Health Monitoring. Small.

[6] Romero F.J., Castillo E., Rivadeneyra A., Toral-Lopez A., Becherer M., Ruiz F.G., Rodriguez N., Morales D.P. (2019). Inexpensive and flexible nanographene-based electrodes for ubiquitous electrocardiogram monitoring. NPJ Flex. Electron..

[7] Yang Y., Gao W. (2019). Wearable and flexible electronics for continuous molecular monitoring. Chem. Soc. Rev..

[8] Trifunovic M., Sberna P.M., Shimoda T., Ishihara R. (2017). Solution-based polycrystalline silicon transistors produced on a paper substrate. NPJ Flex. Electron..

[9] Maiolo L., Pecora A., Maita F., Minotti A., Zampetti E., Pantalei S., Macagnano A., Bearzotti A., Ricci D., Fortunato G. (2013). Flexible sensing systems based on polysilicon thin film transistors technology. Sens. Actuators B Chem..

[10] Subbiah A.S., Mathews N., Mhaisalkar S., Sarkar S.K. (2018). Novel Plasma-Assisted Low-Temperature-Processed SnO2 Thin Films for Efficient Flexible Perovskite Photovoltaics. ACS Energy Lett..

[11] Zhao J., Zhang M., Wan S., Yang Z., Hwang C.S. (2018). Highly Flexible Resistive Switching Memory Based on the Electronic Switching Mechanism in the Al/TiO2/Al/Polyimide Structure. ACS Appl. Mater. Interfaces.

[12] Wang W., Ai T., Yu Q. (2017). Electrical and photocatalytic properties of boron-doped ZnO nanostructure grown on PET–ITO flexible substrates by hydrothermal method. Sci. Rep..

[13] Huang S., Liu Y., Zhao Y., Ren Z., Guo C.F. (2019). Flexible Electronics: Stretchable Electrodes and Their Future. Adv. Funct. Mater..

[14] Khan S., Lorenzelli L., Dahiya R.S. (2015). Technologies for Printing Sensors and Electronics Over Large Flexible Substrates: A Review. IEEE Sens. J..

[15] Nathan A., Ahnood A., Cole M.T., Lee S., Suzuki Y., Hiralal P., Bonaccorso F., Hasan T., Garcia-Gancedo L., Dyadyusha A. (2012). Flexible Electronics: The Next Ubiquitous Platform. Proc. IEEE.

[16] Romero F.J., Rivadeneyra A., Becherer M., Morales D.P., Rodríguez N. (2020). Fabrication and Characterization of Humidity Sensors Based on Graphene Oxide–PEDOT: PSS Composites on a Flexible Substrate. Micromachines.

[17] Goliya Y., Rivadeneyra A., Salmeron J.F., Albrecht A., Mock J., Haider M., Russer J., Cruz B., Eschlwech P., Biebl E. (2019). Next Generation Antennas Based on Screen-Printed and Transparent Silver Nanowire Films. Adv. Opt. Mater..

[18] Falco A., Loghin F.C., Becherer M., Lugli P., Salmerón J.F., Rivadeneyra A. (2019). Low-Cost Gas Sensing: Dynamic Self-Compensation of Humidity in CNT-Based Devices. ACS Sens..

[19] Albrecht A., Salmeron J.F., Becherer M., Lugli P., Rivadeneyra A. (2019). Screen-Printed Chipless Wireless Temperature Sensor. IEEE Sens. J..

[20] Alioto M., Shahghasemi M. (2018). The Internet of Things on Its Edge: Trends Toward Its Tipping Point. IEEE Consum. Electron. Mag..

[21] Wang G., Zhang L., Zhang J. (2012). A review of electrode materials for electrochemical supercapacitors. Chem. Soc. Rev..

[22] Zhong C., Deng Y., Hu W., Qiao J., Zhang L., Zhang J. (2015). A review of electrolyte materials and compositions for electrochemical supercapacitors. Chem. Soc. Rev..

[23] González A., Goikolea E., Barrena J.A., Mysyk R. (2016). Review on supercapacitors: Technologies and materials. Renew. Sustain. Energy Rev..

[24] Pandolfo A.G., Hollenkamp A.F. (2006). Carbon properties and their role in supercapacitors. J. Power Sources.

[25] Lamberti A., Clerici F., Fontana M., Scaltrito L. (2016). A Highly Stretchable Supercapacitor Using Laser-Induced Graphene Electrodes onto Elastomeric Substrate. Adv. Energy Mater..

[26] Peng Z., Lin J., Ye R., Samuel E.L.G., Tour J.M. (2015). Flexible and Stackable Laser-Induced Graphene Supercapacitors. ACS Appl. Mater. Interfaces.

[27] Chen Y., Zhang X., Zhang D., Yu P., Ma Y. (2011). High performance supercapacitors based on reduced graphene oxide in aqueous and ionic liquid electrolytes. Carbon.

[28] Peng Z., Ye R., Mann J.A., Zakhidov D., Li Y., Smalley P.R., Lin J., Tour J.M. (2015). Flexible Boron-Doped Laser-Induced Graphene Microsupercapacitors. ACS Nano.

[29] Wang W., Lu L., Xie Y., Mei X., Tang Y., Wu W., Liang R. (2020). Tailoring the surface morphology and nanoparticle distribution of laser-induced graphene/Co3O4 for high-performance flexible microsupercapacitors. Appl. Surf. Sci..

[30] Shieh J.-Y., Zhang S.-H., Wu C.-H., Yu H.H. (2014). A facile method to prepare a high performance solid-state flexible paper-based supercapacitor. Appl. Surf. Sci..

[31] He D., Marsden A.J., Li Z., Zhao R., Xue W., Bissett M.A. (2018). Fabrication of a Graphene-Based Paper-Like Electrode for Flexible Solid-State Supercapacitor Devices. J. Electrochem. Soc..

[32] Singh R., Tripathi C.C. (2018). Electrochemical Exfoliation of Graphite into Graphene for Flexible Supercapacitor Application. Mater. Today Proc..

[33] Bobinger M.R., Romero F.J., Salinas-Castillo A., Becherer M., Lugli P., Morales D.P., Rodríguez N., Rivadeneyra A. (2019). Flexible and robust laser-induced graphene heaters photothermally scribed on bare polyimide substrates. Carbon.

[34] Romero F.J., Salinas-Castillo A., Rivadeneyra A., Albrecht A., Godoy A., Morales D.P., Rodriguez N. (2018). In-Depth Study of Laser Diode Ablation of Kapton Polyimide for Flexible Conductive Substrates. Nanomaterials.

[35] Yao B., Yuan L., Xiao X., Zhang J., Qi Y., Zhou J., Zhou J., Hu B., Chen W. (2013). Paper-based solid-state supercapacitors with pencil-drawing graphite/polyaniline networks hybrid electrodes. Nano Energy.

[36] Beidaghi M., Gogotsi Y. (2014). Capacitive energy storage in micro-scale devices: Recent advances in design and fabrication of micro-supercapacitors. Energy Environ. Sci..

[37] Du C., Pan N. (2006). High power density supercapacitor electrodes of carbon nanotube films by electrophoretic deposition. Nanotechnology.

[38] Prabaharan S.R.S., Vimala R., Zainal Z. (2006). Nanostructured mesoporous carbon as electrodes for supercapacitors. J. Power Sources.

[39] Hyun W.J., Secor E.B., Hersam M.C., Frisbie C.D., Francis L.F. (2015). High-Resolution Patterning of Graphene by Screen Printing with a Silicon Stencil for Highly Flexible Printed Electronics. Adv. Mater..

[40] Stanford M.G., Zhang C., Fowlkes J.D., Hoffman A., Ivanov I.N., Rack P.D., Tour J.M. (2020). High-Resolution Laser-Induced Graphene. Flexible Electronics beyond the Visible Limit. ACS Appl. Mater. Interfaces.

[41] Torraca P.L., Bobinger M., Romero F.J., Rivadeneyra A., Ricci Y., Cattani L., Morales D.P., Rodríguez N., Salinas-Castillo A., Larcher L. Acoustic characterization of laser-induced graphene film thermoacoustic loudspeakers. Proceedings of the 2019 IEEE 19th International Conference on Nanotechnology (IEEE-NANO).

[42] Lin J., Peng Z., Liu Y., Ruiz-Zepeda F., Ye R., Samuel E.L.G., Yacaman M.J., Yakobson B.I., Tour J.M. (2014). Laser-induced porous graphene films from commercial polymers. Nat. Commun..

[43] Romero F.J., Rivadeneyra A., Toral V., Castillo E., García-Ruiz F., Morales D.P., Rodriguez N. (2018). Design guidelines of laser reduced graphene oxide conformal thermistor for IoT applications. Sens. Actuators A Phys..

[44] Romero F.J., Toral-Lopez A., Ohata A., Morales D.P., Ruiz F.G., Godoy A., Rodriguez N. (2019). Laser-Fabricated Reduced Graphene Oxide Memristors. Nanomaterials.

[45] Ghoniem E., Mori S., Abdel-Moniem A. (2016). Low-cost flexible supercapacitors based on laser reduced graphene oxide supported on polyethylene terephthalate substrate. J. Power Sources.

[46] Wu J.-B., Lin M.-L., Cong X., Liu H.-N., Tan P.-H. (2018). Raman spectroscopy of graphene-based materials and its applications in related devices. Chem. Soc. Rev..

[47] Nguyen V.T., Le H.D., Nguyen V.C., Ngo T.T.T., Le D.Q., Nguyen X.N., Phan N.M. (2013). Synthesis of multi-layer graphene films on copper tape by atmospheric pressure chemical vapor deposition method. Adv. Nat. Sci. Nanosci. Nanotechnol..

[48] Wan Z., Wang S., Haylock B., Kaur J., Tanner P., Thiel D., Sang R., Cole I.S., Li X., Lobino M. (2019). Tuning the sub-processes in laser reduction of graphene oxide by adjusting the power and scanning speed of laser. Carbon.

[49] Karamat S., Sonuşen S., Çelik Ü., Uysallı Y., Özgönül E., Oral A. (2015). Synthesis of few layer single crystal graphene grains on platinum by chemical vapour deposition. Prog. Nat. Sci. Mater. Int..

[50] Lee J.-S.M., Briggs M.E., Hu C.-C., Cooper A.I. (2018). Controlling electric double-layer capacitance and pseudocapacitance in heteroatom-doped carbons derived from hypercrosslinked microporous polymers. Nano Energy.

[51] Yoo J., Kim Y., Lee C.-W., Yoon H., Yoo S., Jeong H. (2017). Volumetric Capacitance of In-Plane- and Out-of-Plane-Structured Multilayer Graphene Supercapacitors. J. Electrochem. Sci. Technol..

[52] Yang D., Bock C. (2017). Laser reduced graphene for supercapacitor applications. J. Power Sources.

[53] Lei Z., Christov N., Zhao X.S. (2011). Intercalation of mesoporous carbon spheres between reduced graphene oxide sheets for preparing high-rate supercapacitor electrodes. Energy Env. Sci..

[54] Oz A., Gelman D., Goren E., Shomrat N., Baltianski S., Tsur Y. (2017). A novel approach for supercapacitors degradation characterization. J. Power Sources.

[55] Senthilkumar S.T., Kalai Selvan R., Lee Y.S., Melo J.S. (2013). Electric double layer capacitor and its improved specific capacitance using redox additive electrolyte. J. Mater. Chem. A.

[56] Patil U.M., Nam M.S., Sohn J.S., Kulkarni S.B., Shin R., Kang S., Lee S., Kim J.H., Jun S.C. (2014). Controlled electrochemical growth of Co(OH)2 flakes on 3D multilayered graphene foam for high performance supercapacitors. J. Mater. Chem. A.

[57] Peng S., Li L., Li C., Tan H., Cai R., Yu H., Mhaisalkar S., Srinivasan M., Ramakrishna S., Yan Q. (2013). In situ growth of NiCo 2 S 4 nanosheets on graphene for high-performance supercapacitors. Chem. Commun..

[58] Kepić D., Sandoval S., Pino Á.P., Del György E., Cabana L., Ballesteros B., Tobias G. (2017). Nanosecond Laser-Assisted Nitrogen Doping of Graphene Oxide Dispersions. ChemPhysChem.

[59] Wu H.D., Zhang Z.H., Barnes F., Jackson C.M., Kain A., Cuchiaro J.D. (1994). Voltage tunable capacitors using high temperature superconductors and ferroelectrics. IEEE Trans. Appl. Supercond..

[60] Molina-Lopez F., Briand D., De Rooij N.F. (2013). Decreasing the size of printed comb electrodes by the introduction of a dielectric interlayer for capacitive gas sensors on polymeric foil: Modeling and fabrication. Sens. Actuators B Chem..

[61] Igreja R., Dias C.J. (2004). Analytical evaluation of the interdigital electrodes capacitance for a multi-layered structure. Sens. Actuators A Phys..

[62] Pech D., Brunet M., Taberna P.-L., Simon P., Fabre N., Mesnilgrente F., Conédéra V., Durou H. (2010). Elaboration of a microstructured inkjet-printed carbon electrochemical capacitor. J. Power Sources.

[63] Lin J., Zhang C., Yan Z., Zhu Y., Peng Z., Hauge R.H., Natelson D., Tour J.M. (2013). 3-Dimensional Graphene Carbon Nanotube Carpet-Based Microsupercapacitors with High Electrochemical Performance. Nano Lett..

[64] Gao W., Singh N., Song L., Liu Z., Reddy A.L.M., Ci L., Vajtai R., Zhang Q., Wei B., Ajayan P.M. (2011). Direct laser writing of micro-supercapacitors on hydrated graphite oxide films. Nat. Nanotechnol..

[65] El-Kady M.F., Kaner R.B. (2013). Scalable fabrication of high-power graphene micro-supercapacitors for flexible and on-chip energy storage. Nat. Commun..

