# Supplementary material for: Comparison of Laser-Synthetized Nanographene-Based Electrodes for Flexible Supercapacitors
Source: Micromachines (Basel). 2020 May 30;11(6):555. doi: 10.3390/mi11060555 (PMC7344853; doi:10.3390/mi11060555)
Supplement: Supplementary file 1 [file micromachines-11-00555-s001.pdf]

micromachines

Supplementary Material: Comparison of Laser-Synthetized Nanographene-Based Electrodes for Flexible Supercapacitors

Francisco J. Romero [1,2,*], Denice Gerardo [3], Raul Romero [1,2], Inmaculada Ortiz-Gomez [4], Alfonso Salinas-Castillo [4], Carmen L. Moraila-Martinez [3], Noel Rodriguez [1,2] and Diego P. Morales [2,5,*]

1 Pervasive Electronics Advanced Research Laboratory, University of Granada, 18071 Granada, Spain; raulromeromad@correo.ugr.es (R.R.); noel@ugr.es (N.R.)
2 Department of Electronics and Computer Technology, University of Granada, 18071 Granada, Spain
3 Parque de Innovación Tecnológica, Facultad de Ciencias Físico Matemáticas, Universidad Autónoma de Sinaloa, 80040 Culiacán, Mexico; denice.gerardo@uas.edu.mx (D.G.); cmorailam@uas.edu.mx (C.L.M.-M.)
4 Department of Analytical Chemistry, Faculty of Science, University of Granada, 18071 Granada, Spain; inmaog@ugr.es (I.O.-G.); alfonsos@ugr.es (A.S.-C.)
5 Biochemistry and Electronics as Sensing Technologies Group, University of Granada, 18071 Granada, Spain
* Correspondence: franromero@ugr.es (F.J.R.); diegopm@ugr.es (D.P.M.); Tel.: +34-958-241-000 (ext. 20193, D.P.M.)

Received: 2 May 2020; Accepted: 29 May 2020; Published: date

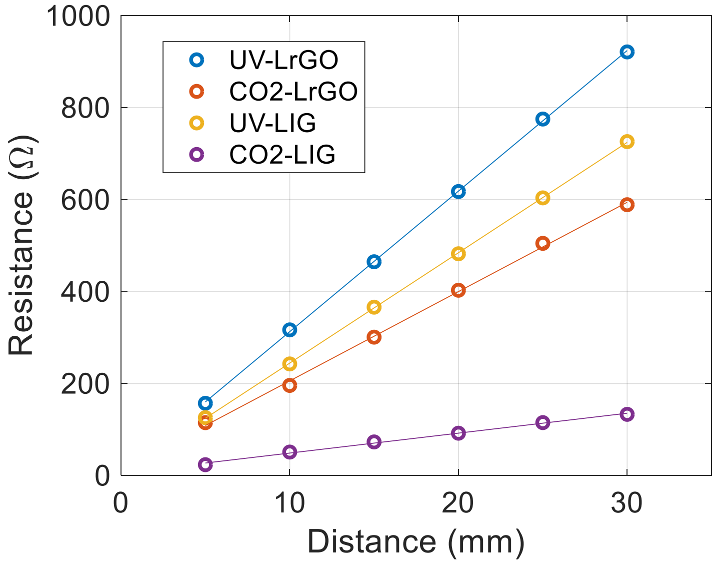

Figure S1. Resistance as a function of the distance between consecutive contacts extracted from TLM measurements [S1].

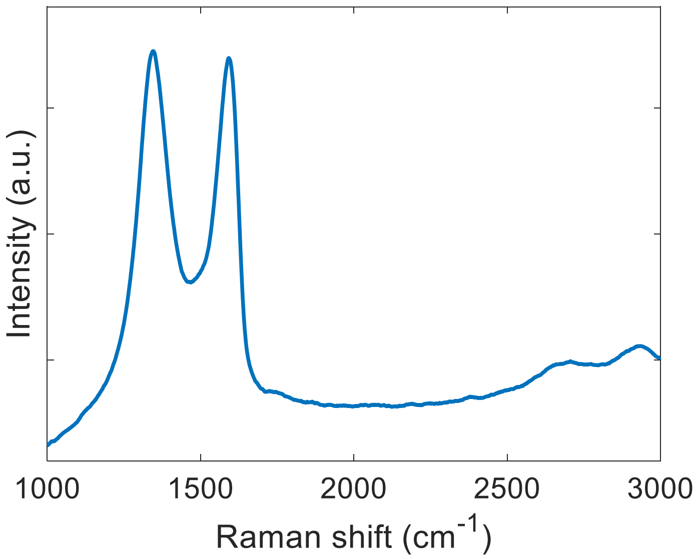

Figure S2. Raman spectra of the graphene oxide. Acquisition parameters: wavelength: 532 nm, data interval: 1 cm^{-1}, exposure time: 15 s, accumulations: 5, center number: 1469.99 cm^{-1}.

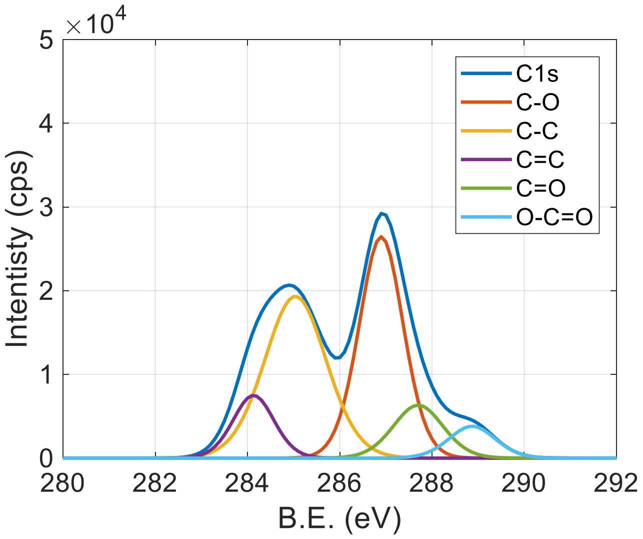

Figure S3. XPS C1s peaks of the graphene oxide.

S1 Romero, F.J.; Rivadeneyra, A.; Ortiz-Gomez, I.; Salinas, A.; Godoy, A.; Morales, D.P.; Rodriguez, N. Inexpensive Graphene Oxide Heaters Lithographed by Laser. *Nanomaterials* **2019**, *9*, 1184, doi:10.3390/nano9091184.
